# Dendrimer-Based Selective Proteostasis-Inhibition Strategy to Control NSCLC Growth and Progression

**DOI:** 10.1371/journal.pone.0158507

**Published:** 2016-07-19

**Authors:** Kyla Walworth, Manish Bodas, Ryan John Campbell, Doug Swanson, Ajit Sharma, Neeraj Vij

**Affiliations:** 1 College of Medicine, Central Michigan University, Mount Pleasant, Michigan, United States of America; 2 Department of Pediatric Respiratory Sciences, The Johns Hopkins School of Medicine, Baltimore, Maryland, United States of America; 3 Department of Chemistry and Biochemistry, Central Michigan University, Mount Pleasant, Michigan, United States of America; Virginia Commonwealth University, UNITED STATES

## Abstract

Elevated valosin containing protein (VCP/p97) levels promote the progression of non-small cell lung carcinoma (NSCLC). Although many VCP inhibitors are available, most of these therapeutic compounds have low specificity for targeted tumor cell delivery. Hence, the primary aim of this study was to evaluate the *in vitro* efficacy of dendrimer-encapsulated potent VCP-inhibitor drug in controlling non-small cell lung carcinoma (NSCLC) progression. The VCP inhibitor(s) (either in their pure form or encapsulated in generation-4 PAMAM-dendrimer with hydroxyl surface) were tested for their *in vitro* efficacy in modulating H1299 (NSCLC cells) proliferation, migration, invasion, apoptosis and cell cycle progression. Our results show that VCP inhibition by DBeQ was significantly more potent than NMS-873 as evident by decreased cell proliferation (p<0.0001, MTT-assay) and migration (p<0.05; scratch-assay), and increased apoptosis (p<0.05; caspase-3/7-assay) as compared to untreated control cells. Next, we found that dendrimer-encapsulated DBeQ (DDN^DBeQ^) treatment increased ubiquitinated-protein accumulation in soluble protein-fraction (immunoblotting) of H1299 cells as compared to DDN-control, implying the effectiveness of DBeQ in proteostasis-inhibition. We verified by immunostaining that DDN^DBeQ^ treatment increases accumulation of ubiquitinated-proteins that co-localizes with an ER-marker, KDEL. We observed that proteostasis-inhibition with DDN^DBeQ^, significantly decreased cell migration rate (scratch-assay and transwell-invasion) as compared to the control-DDN treatment (p<0.05). Moreover, DDN^DBeQ^ treatment showed a significant decrease in cell proliferation (p<0.01, MTT-assay) and increased caspase-3/7 mediated apoptotic cell death (p<0.05) as compared to DDN-control. This was further verified by cell cycle analysis (propidium-iodide-staining) that demonstrated significant cell cycle arrest in the G2/M-phase (p<0.001) by DDN^DBeQ^ treatment as compared to control-DDN. Moreover, we confirmed by clonogenic-assay that DDN^DBeQ^ treatment significantly (p<0.001) inhibits H1299 colony-formation as compared to control/DDN. Overall, encapsulation of potent VCP-inhibitor DBeQ into a dendrimer allows selective VCP-mediated proteostasis-inhibition for controlling NSCLC-tumor growth and progression to allow tumor-targeted sustained drug delivery.

## Introduction

Valosin-containing protein (VCP or p97) is a promising molecular target for anti-cancer drug therapeutics. VCP/p97 is an AAA ATPase molecular chaperone that has been shown to be involved in a variety of different cellular processes including, proliferation, apoptosis, transcription and cell cycle etc [1–7]. VCP regulates these processes by the ubiquitin-proteasome system (UPS). The UPS is a system that manages intracellular levels of all proteins (folded and misfolded) by tagging the proteins with ubiquitin and then transporting these tagged proteins to the proteasome for degradation [1, 4, 8]. Thus, UPS plays a critical role in controlling important cellular mechanisms such as apoptosis, replication and proliferation. Our lab and others have previously shown that cancerous cells have increased levels of VCP, which allows the cancer cells to proliferate and metastasize [1, 2, 4, 8]. Inhibition of this protein’s function has shown promise in decreasing cancerous cellular growth by inducing apoptosis while inhibiting the cell cycle and migration [1–5, 7]. VCP has also been shown to inhibit IκB, which is the endogenous inhibitor of NFκB, a transcription factor that promotes cellular (cancer cell) proliferation and inhibits apoptosis. Thus, increased NFκB levels promote the anti-apoptotic and pro-metastatic abilities the cancerous cell exhibit [1, 2, 4, 9]. There have been many different VCP inhibitors identified with relatively modest potency. Hence, each of these drugs show different efficacy in different cell lines. Some of the strongest VCP/p97 inhibitors (NMS-873 and DBeQ) discovered recently [3, 5, 7, 8, 10] are utilized in this project with an aim to develop a novel anticancer therapeutic. NMS-873 is a noncompetitive inhibitor while DBeQ is an ATP-competitive inhibitor of VCP/p97 [3, 5, 7, 8, 10, 11]. NMS-873 is a very potent and specific inhibitor of VCP that has been shown to activate the unfolded protein response (UPR), interfere with autophagy and induce cancer cell death [7, 8, 10]. Similarly, DBeQ has shown potential in significantly inhibiting vital protein-degradation pathways such as the ERAD (endoplasmic reticulum associated degradation) and the UPS as well as autophagy [1–7]. There are several issues that come with inhibiting VCP in normal non-cancer cells. For instance, VCP is found in all cells and is essential for many healthy cellular processes. If we aim to inhibit this protein, we need to provide sustained and targeted drug delivery. Another issue is that many of the potent VCP inhibitor drugs are not water soluble, and lack adequate specificity for tumor-targeted proteostasis-inhibition. Our lab and others have studied the application of nanodelivery systems to overcome these issues. Several previous studies have looked into utilizing a variety of polymers as nano-drug delivery systems [12–16]. These nano-polymers have been studied in a wide variety of ailments including neurological disorders, cystic fibrosis and various types of cancers [12, 13, 16, 17]. Although, these polymers allow sustained and targeted drug release of water insoluble drugs to become solubilized [12, 13, 17], they have certain limitations for tumor targeting [15, 17]. Hence, this study utilizes a dendrimer that acts similar to the polymers and has an in-built ability to target tumorogenesis, which can be further improved by utilizing specific molecular targets such as VCP [15]. Polyamidoamine dendrimers or PAMAM dendrimers have been extensively studied and have shown substantial potential as a targeted nanodelivery system [15]. These precise nanomaterials can encapsulate a drug and allow its release overtime. Moreover, PAMAM dendrimers (DDN) have been shown to have anti-cancer properties by themselves, even without the encapsulation of anti-cancer drug [15]. Thus, dendrimers encapsulated with an anti-cancer drug are anticipated to be exceptionally potent and tumor-specific. Therefore, this study utilizes a dendrimer encapsulating a potent VCP-inhibitor, DBeQ, to provide a targeted and sustained drug delivery to the non-small cell lung carcinoma cells (NSCLC). Our preliminary results demonstrate that G4-PAMAM dendrimers with encapsulated DBeQ (DDN^DBeQ^) can successfully inhibit VCP proteostasis-function. DDN^DBeQ^ also inhibits H1299 cell proliferation and migration/invasion while increasing apoptosis. Moreover, DDN^DBeQ^ arrests NSCLCs in the G2/M-phase of the cell cycle, providing a proof of concept evidence for future *in vivo* analysis and further development of this novel nano-formulation for controlling NSCLC metastasis.

## Materials and Methods

### Culture Conditions, Transfection and Treatments

H1299 cells [1] were cultured in DMEM/F-12 media supplemented with 10% fetal bovine serum (FBS) and 1% Penicillin, Streptomycin and Amphotericin (PSA) and maintained at 37°C/5% CO_2_ atmosphere. In order to determine the more effective VCP inhibitor, cells were treated with either NMS-873 or DBeQ, at a final concentration of 25μM or 50μM. When utilizing the dendrimers, the final concentration of the drug-treatment was used to set an indicated μM-concentration and equal volumes of dendrimers or PBS were used as controls. In order to visualize ubiquitin-accumulation, cells were transiently transfected with Ubiquitin-RFP plasmid using Lipofectamine^TM^ 2000 reagent (Invitrogen). After 24 hours, cells were treated with either empty dendrimer (DDN), dendrimer-encapsulated DBeQ (DDN^DBeQ^), or PBS (vehicle-control) for 24 hours. Images were captured using the ZOE™ Fluorescent Cell Imager [18].

### Cell Migration Assay

H1299 cells were plated onto a 6-well plate with DMEM/F-12 media containing 10%-FBS and 1% PSA and allowed to grow to ~90% confluence (24 hours). A 10μL pipette tip was used to make a scratch through the middle of the plate. Cells were gently washed (twice) with PBS and fresh media was added to the wells along with the indicated treatments. When comparing the two known potent VCP inhibitors, each well was treated with equimolar concentrations of the inhibitors (NMS-873 or DBeQ, 50μM) or the DMSO control-vehicle. The cells were allowed to migrate for 12 hours and images of the scratch width were taken at 0, 6 and 12 hours after the initial scratch. These images were captured using a Nikon Eclipse TS100 inverted light microscope and a 10x phase objective. The scratch widths were measured using the Infinity Analyze software. The same protocol was utilized when comparing the efficacy of DDN and the DDN^DBeQ^ (50μM) [1].

### Cellular Proliferation Assay

The MTS/MTT (3-(4,5-dimethylthiazol-2-yl)-2,5-diphenyltetrazolium bromide) cell proliferation assay was used to monitor changes in cell growth [1]. H1299 cells were plated into a 96 well plate at 5,000 cells per well. Cells were left overnight to allow adhesion and treated following morning. Fresh media (DMEF/12 +10%-FBS + 1% PSA) was added to each well with NMS-873 (25/50μM), DBeQ (25/50μM) or DMSO-vehicle treatments as indicated. After a 24-hour treatment, 10μL of MTS/MTT reagent (Cell Titer 96^®^ AQ_ueous_ One Solution, Promega) was added to each well. The plate was incubated at 37°C in the CO_2_ incubator for at least 2 hours. After incubation, the plate was read at 490nm on a SpectraMaxM5 microplate reader (Molecular Devices). The same protocol was used for comparing differences in proliferation rates with dendrimer (DDN), DBeQ- encapsulated dendrimer (DDN^DBeQ^, 50μM) or PBS vehicle-control treatment.

### Caspase-3/7 Enzyme Assay

The caspase 3/7 activity was quantified utilizing the Caspase-Glo^®^-3/7 Assay (Promega) [1]. The H1299 cells were seeded on an opaque-bottom 96-well plate (5,000 cells/well) and cultured overnight. The media was replaced and the cells were treated with either NMS-873/DBeQ (25/50μM), or vehicle DMSO-control to obtain a final volume of 100 μL. The cells were treated for 24 hours followed by the addition of 100μL of freshly prepared caspase-3/7 reagent. The plate was incubated at room temperature for 1 hour followed by measurement of changes in luminescence of each well using a SpectraMaxM5 microplate reader (Molecular Devices). The same protocol was used for comparing differences in apoptosis after the dendrimer (DDN), DBeQ encapsulated dendrimer (DDN^DBeQ^, 50μM) or PBS control-vehicle treatment.

### DBeQ Encapsulation in PAMAM Dendrimer and Transmission Electron Microscopy

DBeQ (5 mg, 1.5x10^-5^ mole, 5 equivalents per dendrimer) was first added to a 10 mL round bottom flask with a stir bar followed by 5 mL dimethylsulfoxide at room temperature. Next, we added to this mix a PAMAM dendrimer, DAB core, G = 4, hydroxyl surface (from ethanolamine; MW = 14,305; 46 mg, 3.1x10^-6^ mole) dissolved in 3 mL methanol. This mixture was stirred under nitrogen while sealed with a polypropylene cap for 48 hours at room temperature. The resulting mixture was purified using a Sephadex LH-20 column (10 g of resin in methanol) and eluted with methanol. Fractions containing dendrimer were collected and evacuated using high-speed vacuum to give a constant weight of 40 mg dendrimer (DDN) or dendrimer-DBeQ (DDN^DBeQ^) formulation product. Transmission electron microscopy (TEM) was used to determine the dendrimer size and shape. Briefly, dendrimer were drop-coated on a carbon-coated copper grid for TEM-based size measurement and analysis as recently described [13].

### Transwell Invasion Assay

H1299 cells were seeded and directly treated on a T-25 cell culture flask (Fisher Scientific). Following a 24-hour treatment of either empty dendrimer, dendrimer encapsulated DBeQ (50μM) or vehicle-control PBS, the cells were trypsinized and counted. These treated cells were plated (10,000/well) onto matrigel (Corning, 200μg matrigel per 400μL of serum free media) coated transwell insert (0.4μm pores, Corning). Cells were allowed to migrate for 24 hours under indicated treatments and transwell (blue) were counted using a Nikon Eclipse TS100 inverted light microscope and data is shown as mean ± SEM [1].

### Immunoblotting

H1299 cells were treated on six well plates with either NMS-873 (25μM or 50μM), DBeQ (25μM or 50μM) or left untreated. After 24 hours of treatment, whole cell protein extracts were obtained by adding RIPA buffer, supplemented with 0.5 M EDTA and 1x Halt^TM^ Protease inhibitor cocktail (Thermo Fisher) to each well. The proteins were separated using SDS-PAGE and then immunoblotted onto nitrocellulose membrane. The ubiquitin (Santa Cruz Biotechnology, 1:1000), NFκB (Santa Cruz Biotechnology, 1:1000) and β-actin (equal loading control, Sigma, 1:10,000) antibodies were used as primary antibodies, while goat anti-mouse IgG HRP and goat anti-rabbit IgG HRP were used as a secondary antibodies (1:6000, Amersham). The membranes were visualized using the Clarity™ Western ECL Blotting substrate (Bio-Rad) and C-DiGit Blot Scanner (LI-COR). The same protocol was used when comparing empty-dendrimer (DDN), DBeQ (50μM), dendrimer encapsulated DBeQ (DDN^DBeQ^, 50μM), and vehicle-PBS control with the exception of a longer treatment period (48 hours) in order to better detect long term effects of proteins involved in tumor growth and progression.

### Immunofluorescence Staining and Microscopy

H1299 cells were plated onto a 12-well plate and treated with either dendrimer (DDN), dendrimer encapsulated DBeQ (DDN^DBeQ^, 50μM) or vehicle PBS control. After 24 hours of treatment, cells were fixed using 4%-paraformaldehyde and permeabilized with Triton-X 100, 0.5%). The fixed cells were immunostained with ubiquitin primary antibody (1:500 dilution in 0.5% goat serum, Santa-Cruz Biotechnology). After 1 hour, secondary goat anti-mouse IgG-TR Texas Red antibody (1:1000 dilution in 0.5% goat serum) was added and incubated in the dark for another hour. Hoechst stain (0.5μg/mL) was used to identify nuclei and images were captured using the ZOE™ Fluorescent Cell Imager. The data from Ub-immunostaining and Ubiquitin-RFP transfected cells is shown as mean ± SEM of ubiquitin-positive cells. The same protocol was utilized for the co-staining of Ubiquitin and KDEL. The primary antibodies used were ubiquitin and KDEL (1:500 dilution in 0.5% goat serum, Affinity Bioreagents). The secondary antibodies were goat anti-mouse Texas Red and goat anti-rabbit CFL-488 (1:1000 dilution in 0.5% goat serum).

### Flow Cytometry Based Cell Cycle Analysis

H1299 cells were treated with either NMS-873 (50μM, positive-control), DBeQ (50μM, positive-control), empty dendrimer (DDN), dendrimer encapsulated DBeQ (DDN^DBeQ^, 50μM) or vehicle-control PBS for 24 hours. After treatment, cells were washed with PBS, fixed in ice-cold ethanol (70% v/v) and stored at -20°C overnight. Next morning, cells were washed with PBS (2x) and then re-suspended in propidium iodide stain (PI, 10μg/mL Sigma-Aldrich) with RNase A (20μg/mL, Invitrogen) and BSA (0.1% w/v). The cell suspension was added to a FACS tube and incubated at room temperature in dark for 2 hours. The DNA content of the treated cells were measured using a BD FACS Aria II instrument while the data was analyzed using the BD FACS DIVA software.

### Clonogenic Assay

For standard clonogenic assay, molten-agarose (45°C) was added to complete media (DMEM/F12 with 10% FBS and 1% PSA) at 0.6% final-concentration. This DMEM/F12-agarose mix was quickly pipetted into a 12-well plate and allowed to reach the room temperature. Next, H1299 cells (2.0 x 10^5^ cells/well) were similarly suspended in 0.3%-agarose and were quickly pipetted onto the base agarose layer. This layer was again allowed to completely solidify at room temperature. Wells were then treated with either vehicle-control, DBeQ (50μM, positive-control), DDN or DDN^DBeQ^ (50μM). After the treatment, the plate was kept at 37°C in a CO_2_-incubator till colonies were visible (~4 days). Images were captured using the Nikon Eclipse TS100 inverted light microscope (10x phase objective). The average area of clearly visible colonies were counted and quantified using the Infinity Analyze software.

### Statistical Analysis

Data is represented as mean ± SEM with at least three parallel or independent experimental replicates. Significance was determined using a two-tailed unpaired t-test. A p-value less than 0.05 was considered significant. Densitometry was performed using the Image Studio Digits 4.0 software program as we previously described [1].

## Results

### DBeQ Is a Potent Inhibitor of Cell Migration and Viability in Non-Small Cell Lung Cancer (NSCLC) Cells

**Fig 1:**
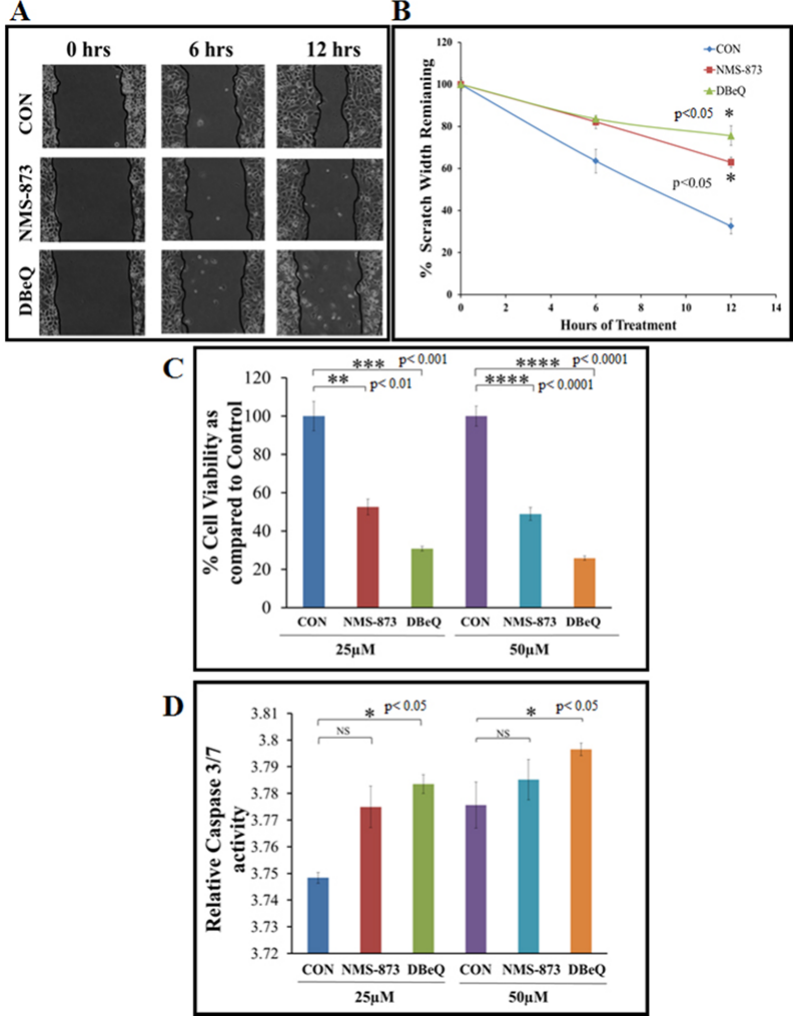
VCP Inhibition by DBeQ effectively controls H1299 proliferation and migration while inducing apoptosis. (A) A uniform scratch was made using a 10μL pipette tip on H1299 confluent six well plates (n = 3). Each well was treated with NMS-873, DBeQ or vehicle-control (DMSO) at 25μM final concentration. Pictures were taken by Infinity Analyze software every 6 hours for 12 hours to quantify changes in migration. (B) The data indicates that DBeQ significantly inhibits the migration of the H1299 cells (p<0.05) as compared to untreated controls or NMS-873 treated cells. (C) H1299 cells were seeded on a 96-well plate and treated with NMS-873 (25/50μM), DBeQ (25/50μM) or DMSO (vehicle-control) for 24 hrs. The Cell Titer AQueous One Solution MTS/MTT reagent (Promega) was added to each well, 1 hour before stopping the experiment and a microplate reader was used to quantify changes in cell viability (n = 5) at the 24-hour time point. Data indicates that DBeQ treatment leads to a more significant (p<0.001) decrease in cell proliferation, as compared to NMS-873. (D) H1299 cells were seeded on a 96-well plate and treated with NMS-873 (25/50μM), DBeQ (25/50μM) or DMSO-control (vehicle). After 24 hours, caspase-3/7 activity was measured using the luminescence caspase-3/7 Glo Assay Kit (Promega). Data shows a significant increase in caspase-3/7 activity with DBeQ treatment as compared to DMSO-control (p<0.05) at both concentrations.

Valosin containing protein (VCP/p97) regulates crucial cellular pathways like cell proliferation, migration and apoptosis [1–7]. We and others have previously shown that elevated VCP protein expression not only correlates with the pathogenesis of NSCLC but also regulates critical mechanisms associated with NSCLC progression and metastasis [1–5, 8, 10, 11, 19]. In this study, we first aimed to compare two potent VCP inhibitors, NMS-873 and DBeQ [3, 5, 7, 8, 10], to determine which of these drugs provide better anti-cancer efficacy, once encapsulated in the dendrimer. To compare the two drugs, we first performed the cell migration (scratch), MTT (proliferation) and capase-3/7 (apoptosis) assays at two different drug concentrations. Both inhibitors decreased the rate of progression of H1299 cells into the scratch as compared to the vehicle control (DMSO) (p<0.05) but DBeQ treatment was more effective in controlling cell migration as compared to NMS-873 (Fig 1A and 1B, p<0.05). To further compare the efficacy of DBeQ and NMS-873 in controlling H1299 cell proliferation [3, 5, 7, 8, 10], a MTS/MTT assay was performed with both drugs at two different concentrations. We found that DBeQ treatment has a significantly greater inhibitory effect on cell proliferation as compared to the vehicle control or NMS-873, at both 25μM (p<0.001) and the 50μM (p<0.0001) concentrations (Fig 1C). Next, we compared the efficacy of the two drugs based on their ability to induce apoptosis of H1299 cells using a caspase-3/7 assay. In a previous study, DBeQ is shown to limit cancer cell growth *via* induction of caspase-mediated cell death [5]. Corroborating with previous report [5] and our proliferation data, we demonstrate here that DBeQ treatment significantly increased caspase-3/7 activity at both 25μM (p<0.05) and 50μM (p<0.05) concentrations as compared to NMS-873 and vehicle-control (DMSO) (Fig 1D). Based on this comparative data, we selected DBeQ for encapsulation in the dendrimer for further evaluation of its efficacy against NSCLC (H1299 cells).

### Dendrimer-Encapsulated DBeQ Inhibits Migration and Invasion of NSCLC

**Fig 2:**
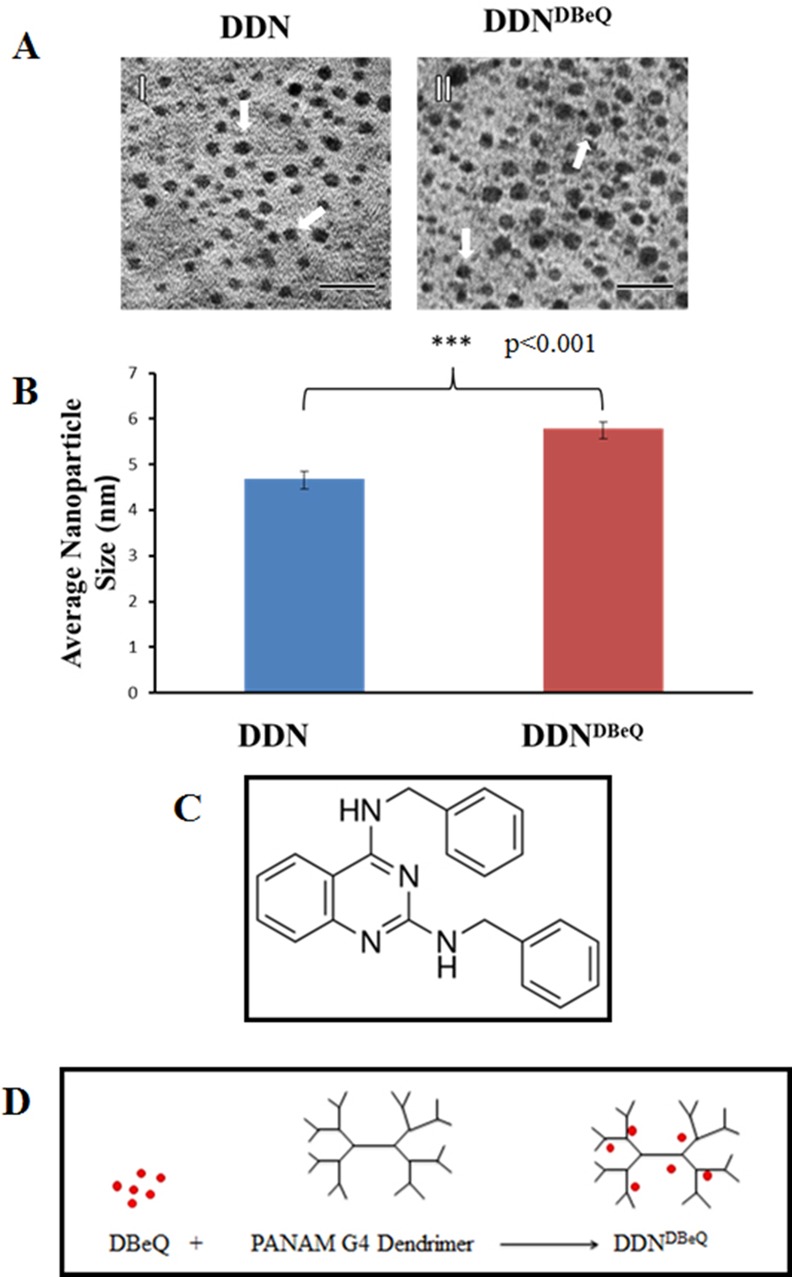
Characterization of dendrimers and DBeQ-loaded dendrimers. (A) Transmission electron microscopy (TEM) images were captured in order to determine the dispersion and size of the empty and DBeQ-loaded dendrimers. (B) QUARTZ PCI TEM analysis software was used to quantify the average diameter of the dendrimers from TEM images. Dendrimer encapsulated DBeQ shows a significant increase in the size of the nanoparticle (p<0.001) indicating that drug was successfully encapsulated. (C) Chemical structure of N², N⁴Dibenzylquinazoline-2,4-diamine (DBeQ, Sigma). (D) Schema showing encapsulation of DBeQ by the dendrimer.

**Fig 3:**
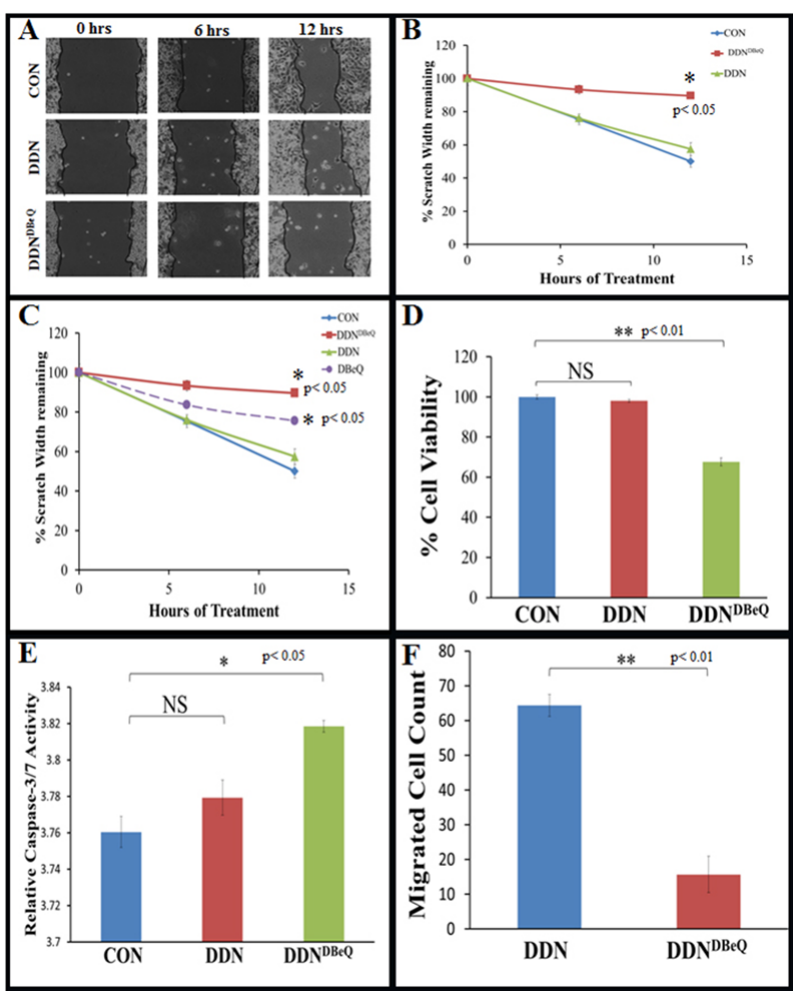
Dendrimer-encapsulated DBeQ significantly inhibits H1299 migration and proliferation while inducing apoptosis. (A) A uniform scratch was made using a 10μL pipette tip on a H1299 confluent six well plate. Each well was treated with dendrimer (DDN), DDN^DBeQ^ and vehicle-control (PBS) at 50μM final concentration for drug (DBeQ) at indicated time points. Pictures were taken by Infinity Analyze software every 6 hours for 12 hours to quantify changes in migration. (B) This data indicates that DDN^DBeQ^ significantly inhibits the migration of the H1299 cells (p<0.05) as compared to DDN or vehicle-control. (C) DBeQ data from Fig 1B was compared with the data in Fig 3B in order to compare the effects of the dendrimer encapsulated DBeQ, DDN^DBeQ^ to direct DBeQ treatment. Data shows DDN^DBeQ^ significantly inhibits the migration of the H1299 cells (p<0.05) as compared to DDN or vehicle-control and is more effective as compared to direct DBeQ treatment. (D) H1299 cells (5,000/well) were seeded on a 96-well plate and treated with Dendrimer (DDN), DDN^DBeQ^ (50μM) or 1x PBS (vehicle-control) for 24 hrs. The Cell Titer AQueous One Solution MTS/MTT reagent was added to each well, 1 hour before stopping the experiment and a microplate reader was used to quantify the H1299 cell viability (n = 5) at the 24 hour time point. Data indicates a significant (p<0.01) decrease in cell proliferation by -inhibition using DDN^DBeQ^ as compared to DDN or vehicle-control. (E) H1299 cells were seeded on a 96-well plate and treated with dendrimer (DDN), DDN^DBeQ^ (50μM) or 1x PBS (vehicle-control). After 24 hours, caspase-3/7 activity was measured using caspase-3/7 Glo luminescence Assay Kit (Promega). Data shows a significant increase in caspase-3/7 activity in DDN^DBEQ^ treated cells as compared to the DDN or vehicle-control (p<0.05). (F) H1299 cells were treated with vehicle-control, DDN or DDN^DBeQ^ (50μM) for 24 hours and transferred to transwell inserts (BD, 0.4μm pores) coated with 200mg Matrigel basement matrix mix for additional 24 hours incubation. Following final incubation, cells that had migrated to the bottom of the membrane were stained with trypan blue and the microscopic field of each insert was visualized using a Nikon light microscope (n = 5, mean ± SEM). The data shows that DDN^DBeQ^ treatment significantly inhibited the invasion of migrating cells as compared to the DDN (p<0.01).

**Fig 4:**
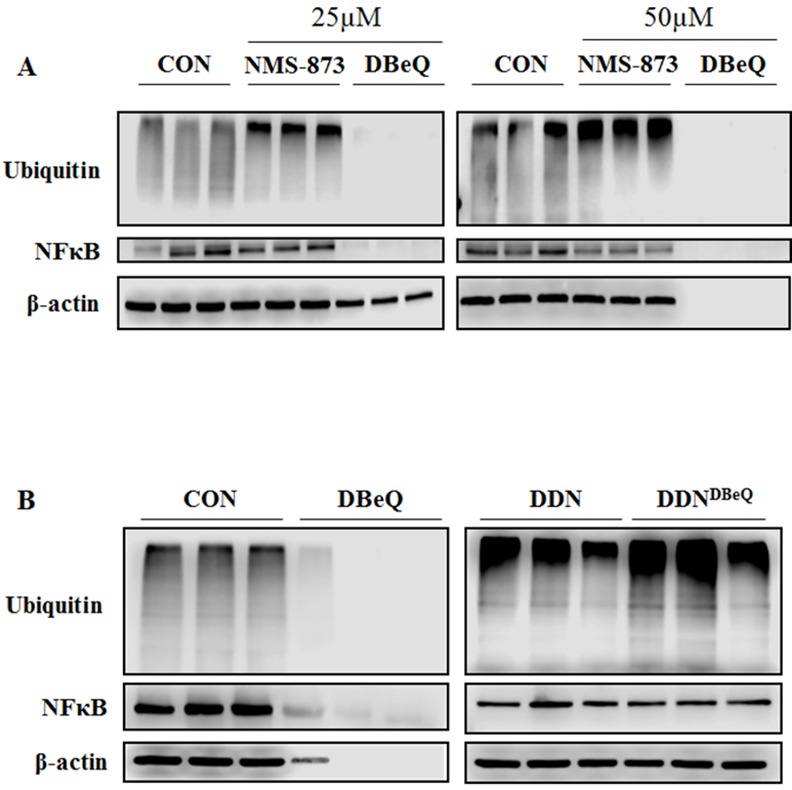
VCP inhibition induces accumulation of ubiquitinated-proteins and decreases NFκB-expression. (A) A western blot analysis of ubiquitin, NFκB and β-actin in soluble protein-fraction of H1299 cells was performed. Cells were seeded on 6-well plates and treated either with NMS-873 (25μM or 50μM), DBeQ (25μM or 50μM) or left untreated for 24 hours. After treatment, cells were lysed and soluble protein fraction prepared for the Western blot. Equal amount of protein samples were separated on 10% SDS-PAGE. The primary antibodies used were a mouse monoclonal ubiquitin/β-actin (1:1000) or a rabbit monoclonal NFκB. Goat anti-mouse HRP (1:6000) or goat anti-rabbit HRP (1:6000) were utilized as secondary antibody. The result indicates that NMS-873 had increased ubiquitinated-protein accumulation with little to no change in NFκB expression with both doses of the treatment. The results indicate that NMS-873 may alter VCP function, thus causing proteostasis-inhibition. The DBeQ immunoblots were not utilized to draw any conclusion on its effect on ubiquitinated-proteins and NFκB-expression due to significant inhibition of the house keeping protein, β-actin in spite of equal loading of total soluble protein. (B) The same protocol was used when comparing vehicle (PBS), DBeQ, empty dendrimer (DDN) or DBeQ-loaded dendrimer (DDN^DBeQ^, 50μM) with the exception of the longer treatment time (48 hours). We observed significant increase in ubiquitinated-protein accumulation in the DDN/DDN^DBeQ^ treated samples as compared to the control-vehicle, although DDN^DBeQ^ was most effective as anticipated. The increase in ubiquitinated-protein accumulation in soluble protein-fraction suggests that the DDN^DBeQ^ treatment alters VCP-function resulting in proteostasis-inhibition and ER-accumulation of these proteins. We also see a significant decrease in expression of tumor-mediator, NFκB in DDN/DDN^DBeQ^ treated samples as compared to the control-vehicle, although DDN^DBeQ^ was most effective as anticipated. The DBeQ immunoblots were not utilized to draw any conclusion on its effect on ubiquitinated-proteins and NFκB-expression due to significant inhibition of the house keeping protein, β-actin, which is used as the loading control.

The potential anticancer drug, DBeQ [3], needs to be selectively targeted to tumor cells since it impacts several housekeeping functions including proteostasis. Hence, DBeQ was encapsulated in a G4-PAMAM dendrimer (DDN^DBeQ^) to develop an effective chemotherapeutic intervention that will not impact normal cells. For encapsulation, DBeQ was added to hydroxyl-terminated generation 4 PAMAM dendrimer. The encapsulated dendrimer-drug complex was purified from free drug by size exclusion chromatography. DBeQ becomes encapsulated and solubilized within the cavities of the dendrimer as shown in Fig 2D. Once the DBeQ was encapsulated, TEM images were captured in order to determine the dispersion and size of the nanoparticles. The images revealed that the dendrimer particles (average size around 6 nm) were larger than a G4-dendrimer, which is approximately 4 nm (Fig 2A and 2B). The nanoparticles that contained the DBeQ were significantly larger (Fig 2A and 2B, p<0.001) implying that the drug was successfully encapsulated into the dendrimer. Next, we compared the potency of dendrimer-encapsulated DBeQ (DDN^DBeQ^) to the empty dendrimer (DDN) based on their ability to modulate cell proliferation, migration and apoptosis. As anticipated, DDN^DBeQ^ treatment significantly inhibited cell migration as compared to control (PBS) or DDN (Fig 3A and 3B, p<0.05) treated H1299 cells. A comparison of cell migration inhibiting potential of DBeQ vs. DDN^DBeQ^ indicates that DDN^DBeQ^ is more effective in inhibiting cellular migration (scratch width) as compared to DBeQ (Fig 3C). Although, DBeQ shows slightly less potency than DDN^DBeQ^ in inhibiting cell migration, its cellular toxicity is much higher, as observed in our other experiments (Fig 4A and 4B), thus making it non-specific for selective tumor cell targeting. Key to developing an effective cancer drug formulation is an effective control of tumor growth by a non-toxic compound, properties that are difficult to achieve with a free drug. Thus, dendrimer based tumor-targeted DBeQ delivery can allow development of an effective chemotherapeutic intervention that will not affect normal cells. In further analysis, DDN^DBeQ^ treatment significantly decreased cell proliferation (Fig 3D, p<0.01), while increasing caspase-3/7 activity, as compared to the control (PBS) or DDN (Fig 3E, p<0.05). To further validate the potential of DDN^DBeQ^ in inhibiting cancer cell progression, a transwell cell invasion model was used to quantify changes in tumor (H1299) cell migration/invasion through a matrigel matrix, after treatment with either DDN or DDN^DBeQ^. The data shows that the number of migrated cells (trypan-blue positive cells) was significantly decreased upon treatment with DDN^DBeQ^ as compared to DDN-control (Fig 3F, p<0.01). These results suggest that treatment with DDN^DBeQ^ inhibits the NSCLC proliferation and migration (metastatic potential), while inducing its apoptosis, indicating its potency as a therapeutic candidate to restrict NSCLC growth and progression.

### Dendrimer-Based VCP-Inhibition Induces Accumulation of Ubiquitinated-Proteins and Inhibits NFκB-Expression

**Fig 5:**
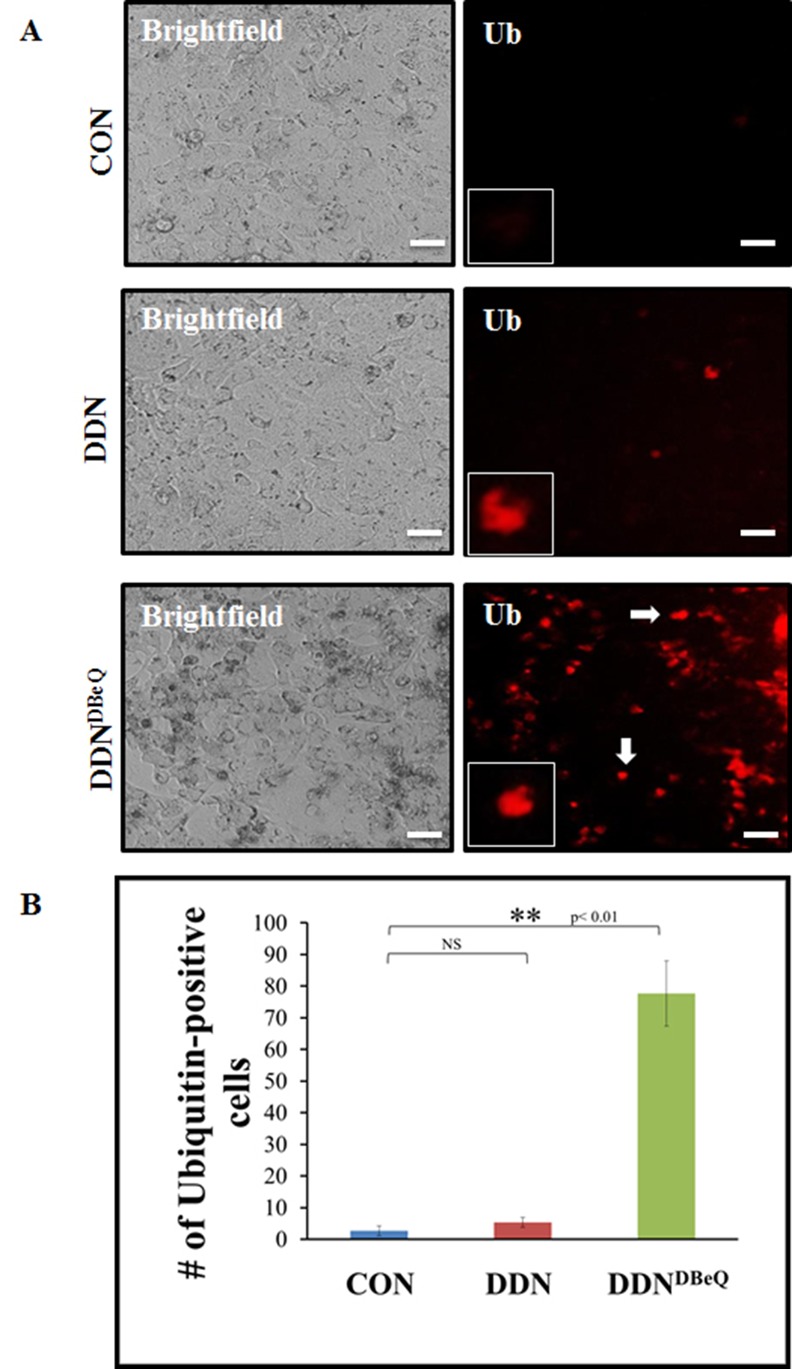
The number of ubiquitin-positive cells significantly increases with DDN^DBEQ^ treatment. H1299 cells were plated on a 12-well plate and transfected with RFP-ubiquitin. After 24 hours, the cells were treated with control-vehicle (PBS), dendrimer (DDN) or DBeQ-encapsulated dendrimer (DDN^DBeQ^, 50μM). ZOE™ Fluorescent Cell Imager was used to capture bright-field and fluorescent images after 48 hours of transfection. Scale bars = 56μm. The results show a significant increase in the number of ubiquitin-positive cells upon DDN^DBeQ^ treatment as compared to the control-vehicle or DDN (p<0.01). The ubiquitinated-protein accumulation in DDN^DBeQ^ treated cells is primarily in areas around nucleus suggesting ER-accumulation of these proteins.

**Fig 6:**
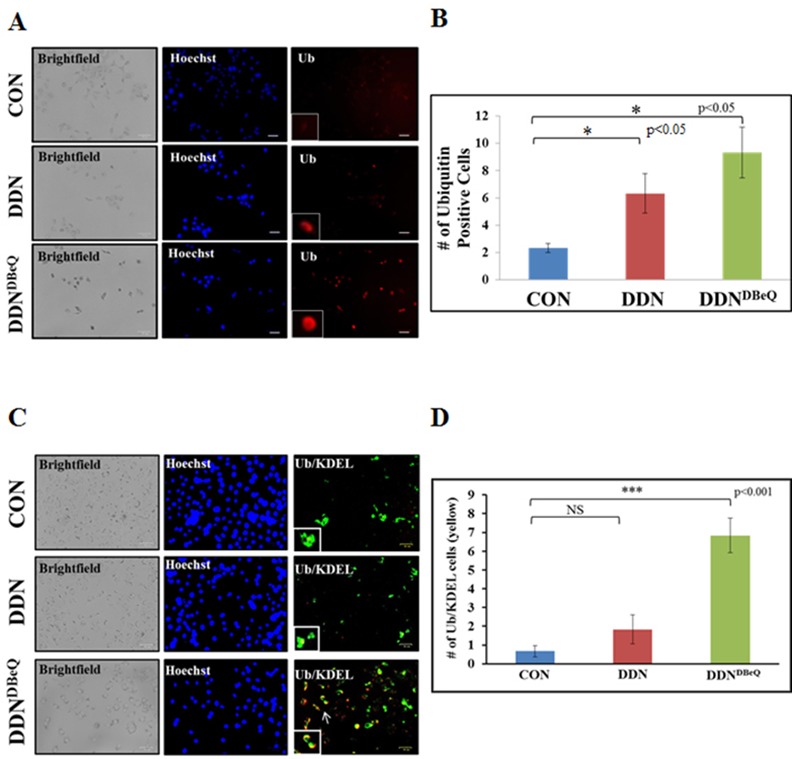
DDN^DBeQ^ treatment significantly increases the accumulation and ER-localization of ubiquitinated-proteins. (A) H1299 cells were plated onto a 12-well plate and treated with the vehicle-control (PBS), dendrimer-control (DDN) or the DBeQ-encapsulated dendrimer (DDN^DBeQ^, 50μM). After 24 hours exposure, the cells were fixed using the 4%-paraformaldehyde and then stained with the primary antibody (1:1000, Ub, mouse monoclonal) followed by a secondary antibody (1:1000, goat anti-mouse IgG Texas Red). Hoechst staining (blue) was used to localize the nuclei. ZOE™ Fluorescent Cell Imager was used to capture brightfield and fluorescent images where scale bars represent 56μm in length. (B) This data shows an increase in ubiquitinated-protein accumulation in the cells treated with DDN^DBeQ^ as compared to vehicle/DDN-control. These results suggest that dendrimer-encapsulated DBeQ is effective in inhibiting VCP function within the cell causing increased accumulation of ubiquitinated-proteins (potentially in ER) by selective proteostasis-inhibition. (C) H1299 cells were plated onto a 12-well plate and treated with the vehicle-control (PBS), dendrimer-control (DDN) or the DBeQ-encapsulated dendrimer (DDN^DBeQ^, 50μM). After, 24 hours of treatment, cells were fixed using 4%-paraformaldehyde and then stained with the primary antibodies (1:1000, Ub, Mouse monoclonal and 1:1000, KDEL, rabbit polyclonal) followed by secondary antibodies (1:1000, goat anti-mouse IgG Texas Red and 1:1000, goat anti-rabbit IgG CFL-488). ZOE™ Fluorescent Cell Imager was used to capture bright-field and fluorescent images to identify changes in expression and localization of ubiquitinated-proteins with DDN^DBeQ^ treatment. (D) The number of Ub/KDEL co-stained cells (yellow) were quantified for each treatment group. The results show a significant increase in accumulation and co-localization of ubiquitinated-proteins with an ER marker, in the DDN^DBeQ^ treatment group as compared to the control PBS-vehicle or DDN treatments (p<0.001). These results confirm that the DDN^DBeQ^ causes accumulation of ubiquitinated-proteins in ER suggesting selective VCP mediated proteostasis-inhibition.

Elevated VCP levels are known to be associated with increased cell survival and metastasis of NSCLC cells [1, 2, 4] *via* an increased NFκB-mediated pro-survival mechanism [1, 2, 4, 9]. This is attributed to an increased ubiquitin-dependent proteasomal degradation (component of proteostasis) of IκB, an inhibitor of NFκB. We postulated that potent VCP-inhibition using both NMS-873 or DBeQ (25μM or 50 μM) would block VCP-mediated proteostasis that will not only lead to NFκB-inhibition but also hamper NSCLC cell growth and invasion due to aggregation of ubiquitinated-proteins in the endoplasmic reticulum (ER). As anticipated, we found that treatment of H1299 cells with both concentrations of NMS-873 leads to accumulation of ubiquitinated-proteins in the soluble protein-fraction (potentially ER), as compared to the untreated control (Fig 4A), implying proteostasis-inhibition [1, 20]. There is also a slight decrease in the NFκB expression when treated with NMS-873 as compared to the untreated control. DBeQ proved to be exceptionally toxic to the H1299 cells by 24hrs of treatment (Fig 4A), which gets worse with longer 48hrs of treatment (Fig 4B) as seen by significant decrease or absence of a house keeping protein, β-actin in spite of equal total protein loading. A significant accumulation of ubiquitinated-proteins and decreased levels of the critical metastatic mediator NFκB was also observed in DDN^DBeQ^ treated H1299 cells (Fig 4B) as compared to control/DDN. These results suggest that DDN^DBeQ^ mediated VCP/proteostasis-inhibition might be the underlying mechanism behind the observed decrease in cell proliferation and migration/invasion, and increased apoptotic cell death in DDN^DBeQ^ treated NSCLC cells. In order to further confirm these findings, H1299 cells were transfected with ubiquitin-RFP for 24 hrs and then treated with PBS (control), DDN or DDN^DBeQ^ followed by fluorescence microscopy to quantify the changes in number of ubiquitin-RFP positive cells. We found that accumulation ubiquitinated-proteins (red) in DDN^DBeQ^ treated cells seems to be primarily localized in ER due to its proximity to the nuclei. Our data demonstrates that DDN^DBeQ^ treatment leads to an increase in the number of ubiquitin-positive cells (Fig 5A and 5B, p<0.01) validating that VCP inhibition negatively impacts proteostasis, thereby potentially controlling NSCLC progression. These results were further confirmed using ubiquitin-immunostaining and fluorescence microscopy of H1299 cells treated with PBS (control), DDN or DDN^DBeQ^ for 24 hours. The data verifies our transfection and immunoblotting results and shows that DDN^DBeQ^ treated cells had significantly elevated ubiquitin-accumulation (Fig 6A and 6B, p<0.05). We also confirmed that the ubiquitinated-protein accumulation is occurring within the ER by co-staining with an ER-marker, KDEL. We observe significantly higher numbers of Ub-KDEL positive cells (Fig 6C and 6D, p<0.001) in DDN^DBeQ^ treated cells as compared to the control/DDN groups, verifying ER accumulation of ubiquitinated-proteins as anticipated due to VCP-mediated proteostasis dysfunction. Overall, these results suggest that DDN^DBeQ^ treatment effectively inhibits VCP function as seen by selective proteostasis obstruction (ubiquitin-accumulation). We postulate VCP mediated proteostasis-inhibition as a potential mechanism for DDN^DBeQ^ mediated control of NSCLC growth and progression.

### Dendrimer Mediated VCP-Inhibition Arrests Cell Cycle Progression of NSCLC

**Fig 7:**
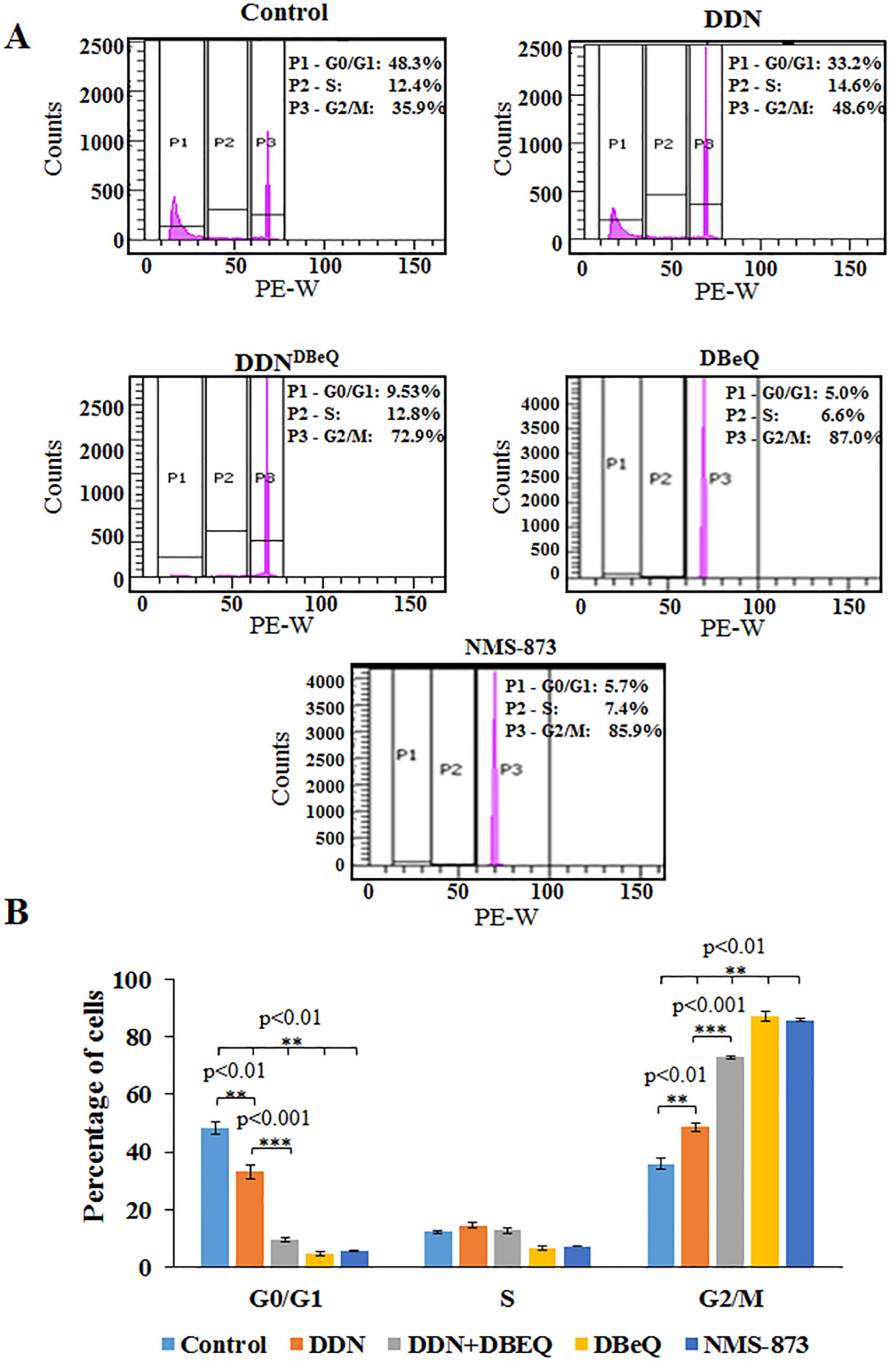
Selective VCP inhibition induces significant cell cycle arrest in G2/M phase. (A) Following a 24-hour treatment period with control-PBS, NMS-873/DBeQ (50μM, positive-control), dendrimer (DDN) or dendrimer-encapsulated DBeQ (DDN^DBeQ^, 50 μM), cells were fixed with ice-cold 70%-ethanol followed by staining with propidium iodide (10μg/mL) for 1 hour. The DNA content of the treated cells was captured using the BD FACS Aria II instrument while the data was analyzed using the BD FACS DIVA software. (B) Our results indicate that there is significant cell cycle arrest in the G2/M phase when treated with dendrimer-encapsulated DBeQ (DDN^DBeQ^) (p<0.001) as compared to controls (PBS/DDN). As anticipated, NMS-873 and DBeQ controls (positive) also show significant cellular arrest in the G2/M phase as compared to the vehicle-control (PBS) (p<0.01). Data shows that DDN^DBeQ^ treatment is effective in selectively inhibiting VCP-function resulting in the decrease in the number of cells undergoing mitosis as seen by significant increase in G2/M arrest.

We have previously shown that VCP inhibition by shRNA or small molecule inhibitor (Eer1) causes cell cycle arrest at the G0/G1 phase (~1.25 fold, control *vs* shRNA/Eer1) in H1299 cells [1]. Here, we investigated the impact of two different VCP inhibitors (NMS-873 and DBeQ) and a dendrimer-encapsulated VCP-inhibitor (DDN^DBeQ^) on H1299 cell cycle progression by utilizing propidium iodide (PI) staining, as described earlier [1]. First, we analyzed the VCP inhibitors as positive controls and found that both inhibitors caused cell cycle arrest in the G2/M phase of the cell cycle (Fig 7A and 7B) as compared to untreated control. We then tested the efficacy of dendrimer-DBeQ formulation (DDN^DBeQ^) designed for tumor targeting and found that there is a similar significant cell cycle arrest in the G2/M phase (Fig 7A and 7B, p<0.001) with DDN^DBeQ^ treatment (~1.5–2.03 fold; DDN^DBeQ^ *vs* DDN/PBS-controls) as compared to the DDN/PBS-controls. The DDN^DBeQ^ treatment had 72.9% cells arrested in the G2/M phase while the DDN-control (48.6%) and PBS-control (35.9%) group had much lower percentages of cells within this phase. Moreover, the data also demonstrates that the increase in number of cells in the G2/M phase corresponds to a significant decrease in the number of cells in the G0/G1 phase. The current data as compared to previous findings using VCP shRNA or Eer1 [1] indicate a higher potency of current VCP targeting strategy (DBeQ/NMS-873 or DDN^DBeQ^). In conclusion, our present data verifies the vital role of VCP in cell cycle progression of H1299 cells verifying its crucial function in promoting tumor growth. Thus, selectively inhibiting VCP’s functions can be developed as potent therapeutic strategy to control NSCLC growth and metastasis, which warrants further standardization in pre-clinical murine models to ensure tumor specific drug delivery.

### Selective VCP Mediated Proteostasis-Inhibition Controls NSCLC Colony-Formation

**Fig 8:**
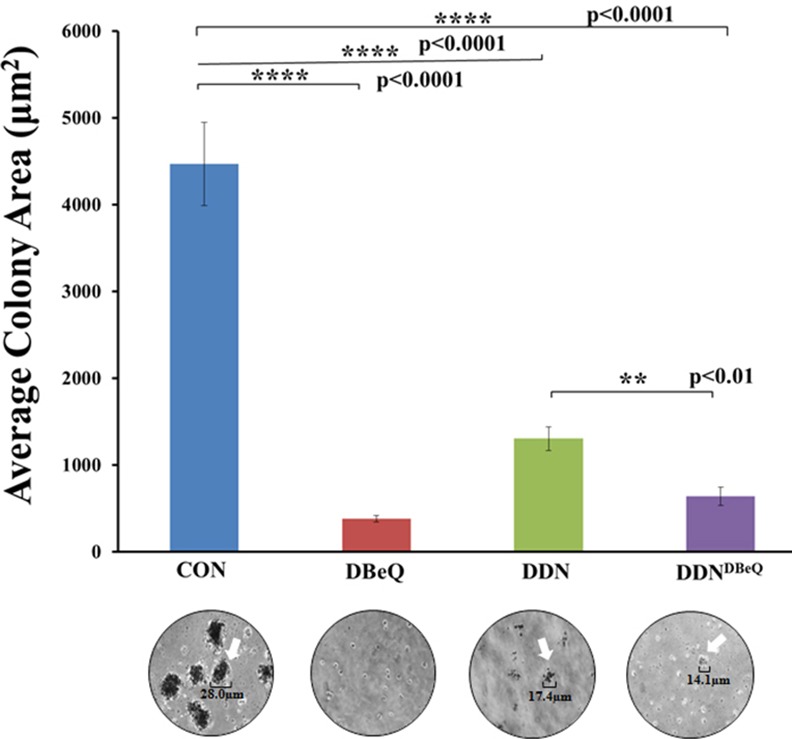
Selective VCP mediated proteostasis-inhibition controls the growth of NSCLC colonies. H1299 cells were suspended in serum containing media with 0.3% agarose and plated over a 0.6% agarose base layer on a 12-well plate (2.0 x10^5^/well) (n = 3). Fresh media with control-PBS-vehicle, empty-dendrimer (DDN), DBeQ (50μM, positive control) or DBeQ-encapsulated dendrimer (DDN^DBeQ^, 50μM) was added to the top of the cells suspended in agarose. The plate was kept in a 37°C incubator until colonies were visible and pictures were captured using a Nikon Eclipse TS100 inverted light microscope at 10x phase objective magnification. The average number and area of colonies were quantified using the Infinity Analyze software. The data indicates that DBeQ, DDN and DDN^DBeQ^ treatments significantly decrease the number and area of NSCLC colonies as compared to PBS-vehicle control (p<0.001). These results support our findings that dendrimer encapsulated DBeQ is effective in limiting NSCLC growth suggesting its potential in controlling tumor progression and metastasis.

We next investigated if VCP mediated proteostasis-inhibition impacts NSCLC (H1299) colony formation by performing a clonogenic assay. Briefly, H1299 cells (2.0 X 10^5^) were seeded on a 12-well plate on the two layers of agarose (bottom base layer-0.6%, top cell layer-0.3%). Next, cells were treated with either control-PBS vehicle, DBeQ (positive-control), DDN or DDN^DBeQ^ and images were captured as discussed above in the methods. Our results indicate that DBeQ and DDN/DDN^DBeQ^ effectively inhibit H1299 colony formation as compared to the control (Fig 8, p<0.0001). Although, DBeQ/DDN^DBeQ^ were most effective, as discussed above, we anticipate that DBeQ treatment is toxic to the cells while DDN^DBeQ^ can allow effective chemotherapeutic intervention of NSCLC without affecting the normal cells. In summary, these results indicate that DDN^DBeQ^ has the potential to provide sustained drug delivery to tumor cells by selectively inhibiting VCP mediated proteostasis.

## Discussion

We and others have identified valosin-containing protein (VCP/p97 AAA-ATPase) as a promising therapeutic target for non-small cell lung cancer (NSCLC) and several other types of cancers [1, 2, 4, 10], as it regulates critical protein-homeostasis mechanisms to control levels of multiple cellular pathways such as proliferation, migration, inflammation and apoptosis etc [1–7, 21]. Hence, several VCP-inhibitors have been developed with a goal to allow therapeutic intervention but the concern with most of these VCP inhibitors is their low potency [3] and significant toxicity, which can lead to non-specific activity on normal cells. In order to develop an effective cancer drug formulation, one has to achieve effective control of tumor growth while making sure compound is non-toxic, although it is difficult to achieve these properties with a free drug. Hence, we utilized here a dendrimer-encapsulation of a potent VCP inhibitor drug, DBeQ, in order to circumvent this problem and allow tumor-specific targeting by using a sustained and targeted drug release system. We demonstrate that dendrimer-encapsulated DBeQ (DDN^DBeQ^) shows substantial promise in controlling NSCLC (H1299) cell proliferation and migration, while inducing cell cycle arrest and caspase-3/7 activity. We further validate proteostasis-inhibition (ubiquitin accumulation) as the underlying mechanism of DDN^DBeQ^ mediated obstruction of NSCLC growth and progression.

Our group and others have previously discussed and reported the critical pathogenic role of VCP in neurological disorders, cystic fibrosis (CF), chronic obstructive pulmonary disease (COPD) and various forms of cancers including NSCLC [1, 2, 4, 22–24]. We have reported the *in vitro* and *in vivo* anti-cancer potential of inhibiting VCP expression or function using si/sh-RNA or small-molecule drugs such as Eeyarestatin I (EerI) in our earlier study [1]. As discussed before, VCP is crucial for maintaining normal protein homeostasis, as it is involved in the degradation of misfolded proteins *via* ERAD (endoplasmic reticulum associated protein degradation) or a peri-nuclear autophagy that mediates cytosolic clearance of aggresomes [4, 10, 11]. Cancer cells are rapidly dividing and metabolically more active than normal cells, thus requiring a more robust proteostasis mechanism. This is achieved by a significantly higher expression of VCP in a myriad variety of cancer cells, including NSCLC [1, 2, 4, 10, 22]. Therefore, VCP inhibiting drugs need to specifically target cancerous cells while not impacting normal cells where optimal VCP-function is essential. As discussed above, although many VCP inhibitors are available, they are not useful therapeutics due to their un-specificity [3] and toxicity. These factors pose significant challenge in using VCP as a target for anti-cancer therapeutics. Hence, in this study we synthesized a dendrimer-based nano-drug formulation to increase specificity and provide sustained targeted-delivery of potent VCP inhibitor to the cancer cells. We first sought to determine which VCP inhibitor would provide the most significant inhibitory effect on NSCLC (H1299 cells). To this end, we tested two potent VCP inhibitors; NMS-873, a non-competitive inhibitor of VCP function, and DBeQ, an ATP-competitive inhibitor, for their potency against H1299 cells. Our preliminary results show that although both drugs controlled cell migration and proliferation, and enhanced caspase 3/7-mediated apoptosis. Moreover, NMS-873 increased the accumulation of ubiquitinated-proteins and decreased NFκB protein expression in H1299 cells. Intriguingly, DBeQ treatment causes excessive cellular toxicity as seen by significant decrease in the expression of the housekeeping protein, β-actin. Based on our overall results where DBeQ demonstrates better efficacy in the functional assays as compared to NMS-873, we selected DBeQ for encapsulation into the dendrimer to improve its effectiveness in selectively targeting tumor cells and allowing controlled drug release for selective VCP mediated proteostasis-inhibition of NSCLC.

Dendrimer-based therapeutics have gained substantial success in tumor-targeting and sustained drug delivery in variety of cancers, including NSCLC [15, 25–27]. In one study, a tumor-specific peptide conjugated to a G4-PAMAM dendrimer, was described as a potential drug carrier with tumor-specific targeting characteristics [25]. Here, we first needed to evaluate whether the dendrimer-encapsulated DBeQ (DDN^DBeQ^) impeded NSCLC cell migration and proliferation, while also increasing apoptosis. We report here that DDN^DBeQ^ formulation not only decreased the toxicity of DBeQ but also provide a potent inhibition of cellular proliferation and migration while inducing NSCLC (H1299 cells) apoptosis. Our data highlights the potential of DDN^DBeQ^ in providing tumor-targeted delivery of VCP inhibitor(s) to control NSCLC growth, progression and metastasis. We also investigated the underlying mechanisms of DDN^DBeQ^ mediated impedance of NSCLC cell growth. One of the plausible mechanisms that could direct the anti-cancer effects of DDN^DBeQ^ is elevated ER-stress. VCP inhibition leads to ER-overload, which if not restricted triggers ER stress [4, 5, 8, 19] and thus the activation of the apoptotic arm of the unfolded protein response (UPR), leading to initiation of cell death pathways [4, 5, 8, 11, 19, 28]. Our data supports this, as we not only report significant increase in ubiquitin-accumulation but also elevated apoptosis in DDN^DBeQ^ treated H1299 cells as compared to controls. Thus, we concluded that DDN^DBeQ^ mediates selective VCP/proteostasis-inhibition as evident from the increased levels of poly-ubiquitinated proteins in soluble (ER) protein-fraction instead of insoluble (cytosol, aggresome-bodies).

Given the prominent role of VCP in regulating both cellular proliferation and apoptosis, we next postulated that inhibiting VCP might modulate the cell cycle. Our data supports this notion and shows that NMS-873/DBeQ or DDN^DBeQ^ mediated inhibition of VCP function causes a significant cell cycle arrest in the G2/M phase. As anticipated, data also demonstrates an increase in number of cells in the G2/M phase that corresponds to a significant decrease in the number of cells in the G0/G1 phase of a particular treatment group. Numerous studies have shown the importance of G2/M cell cycle arrest for anti-cancer therapeutics, as G2/M- arrest indicates the cell cycle arrest during the progression into mitosis that leads to programmed tumor cell death or apoptosis [29–31]. The present data shows that DDN^DBeQ^ effectively arrests the NSCLC cell progression in G2/M phase while retaining selective VCP-mediated proteostasis-inhibition (accumulation of ubiquitinated-proteins) property and its potency in controlling NFκB-inhibition activity, and tumor cell cycle, growth and invasion without inducing overall cellular toxicity as seen with DBeQ. Moreover, proposed nano-formulation is more effective (DDN^DBeQ^) as compared to other VCP inhibiting compounds such as Eer1, which showed only a minimal cell cycle arrest in the G0/G1 phase [1, 5, 32].

Overall, our findings not only support previous studies [1–5, 8, 10, 11, 19] but also provide substantial evidence that selective dendrimer-based VCP-inhibition is a promising therapeutic strategy for controlling the NSCLC progression. Moreover, the proposed strategy has a potential for potent tumor-targeted VCP-inhibition. This is the first report showing that encapsulation of a potent VCP inhibitor, DBeQ into a G4-PAMAM dendrimer, not only improves the specificity of inhibitor but also decreases the toxicity while retaining the potency. Thus, the DDN^DBeQ^ formulation provides significant benefits over unconjugated VCP inhibitor drugs, to control NSCLC progression and has the potential for further development to allow tumor-targeted sustained drug delivery.

## References

[1] Valle CW, Min T, Bodas M, Mazur S, Begum S, Tang D, et al Critical role of VCP/p97 in the pathogenesis and progression of non-small cell lung carcinoma. PloS one. 2011;6(12):e29073 Epub 2012/01/05. 10.1371/journal.pone.0029073 22216170 PMC3245239

[2] Fessart D, Marza E, Taouji S, Delom F, Chevet E. P97/CDC-48: proteostasis control in tumor cell biology. Cancer Lett. 2013;337(1):26–34. 10.1016/j.canlet.2013.05.030 . 23726843

[3] Fang CJ, Gui L, Zhang X, Moen DR, Li K, Frankowski KJ, et al Evaluating p97 inhibitor analogues for their domain selectivity and potency against the p97-p47 complex. ChemMedChem. 2015;10(1):52–6. 10.1002/cmdc.201402420 25377500 PMC4280364

[4] Vij N. AAA ATPase p97/VCP: cellular functions, disease and therapeutic potential. Journal of cellular and molecular medicine. 2008;12(6A):2511–8. 10.1111/j.1582-4934.2008.00462.x 18798739 PMC4514128

[5] Chou TF, Brown SJ, Minond D, Nordin BE, Li K, Jones AC, et al Reversible inhibitor of p97, DBeQ, impairs both ubiquitin-dependent and autophagic protein clearance pathways. Proc Natl Acad Sci U S A. 2011;108(12):4834–9. 10.1073/pnas.1015312108 21383145 PMC3064330

[6] Meyer H, Weihl CC. The VCP/p97 system at a glance: connecting cellular function to disease pathogenesis. J Cell Sci. 2014;127(Pt 18):3877–83. 10.1242/jcs.093831 25146396 PMC4163641

[7] Chou TF, Bulfer SL, Weihl CC, Li K, Lis LG, Walters MA, et al Specific inhibition of p97/VCP ATPase and kinetic analysis demonstrate interaction between D1 and D2 ATPase domains. J Mol Biol. 2014;426(15):2886–99. 10.1016/j.jmb.2014.05.022 24878061 PMC4102644

[8] Parzych K, Chinn TM, Chen Z, Loaiza S, Porsch F, Valbuena GN, et al Inadequate fine-tuning of protein synthesis and failure of amino acid homeostasis following inhibition of the ATPase VCP/p97. Cell Death Dis. 2015;6:e2031 10.1038/cddis.2015.373 26720340 PMC4720905

[9] Dai RM, Chen E, Longo DL, Gorbea CM, Li CC. Involvement of valosin-containing protein, an ATPase Co-purified with IkappaBalpha and 26 S proteasome, in ubiquitin-proteasome-mediated degradation of IkappaBalpha. J Biol Chem. 1998;273(6):3562–73. . 9452483 10.1074/jbc.273.6.3562

[10] Magnaghi P, D'Alessio R, Valsasina B, Avanzi N, Rizzi S, Asa D, et al Covalent and allosteric inhibitors of the ATPase VCP/p97 induce cancer cell death. Nat Chem Biol. 2013;9(9):548–56. 10.1038/nchembio.1313 . 23892893

[11] Chou TF, Deshaies RJ. Development of p97 AAA ATPase inhibitors. Autophagy. 2011;7(9):1091–2. 21606684 10.4161/auto.7.9.16489 PMC3210319

[12] Kobes JE, Daryaei I, Howison CM, Bontrager JG, Sirianni RW, Meuillet EJ, et al Improved Treatment of Pancreatic Cancer With Drug Delivery Nanoparticles Loaded With a Novel AKT/PDK1 Inhibitor. Pancreas. 2016 10.1097/MPA.0000000000000607 . 26918875 PMC4983222

[13] Vij N, Min T, Marasigan R, Belcher CN, Mazur S, Ding H, et al Development of PEGylated PLGA nanoparticle for controlled and sustained drug delivery in cystic fibrosis. Journal of nanobiotechnology. 2010;8:22 10.1186/1477-3155-8-22 20868490 PMC2954907

[14] Upadhaya SK, Swanson DR, Tomalia DA, Sharma A. Analysis of polyamidoamine dendrimers by isoelectric focusing. Anal Bioanal Chem. 2014;406(2):455–8. 10.1007/s00216-013-7458-0 . 24247550

[15] Yang H. Targeted nanosystems: Advances in targeted dendrimers for cancer therapy. Nanomedicine. 2016;12(2):309–16. 10.1016/j.nano.2015.11.012 26706410 PMC4789125

[16] Bisht S, Feldmann G, Soni S, Ravi R, Karikar C, Maitra A. Polymeric nanoparticle-encapsulated curcumin ("nanocurcumin"): a novel strategy for human cancer therapy. J Nanobiotechnology. 2007;5:3 10.1186/1477-3155-5-3 17439648 PMC1868037

[17] Roy I, Vij N. Nanodelivery in airway diseases: challenges and therapeutic applications. Nanomedicine: nanotechnology, biology, and medicine. 2010;6(2):237–44. 10.1016/j.nano.2009.07.001 19616124 PMC2847663

[18] Shivalingappa PC, Hole R, Westphal CV, Vij N. Airway Exposure to E-Cigarette Vapors Impairs Autophagy and Induces Aggresome Formation. Antioxidants & redox signaling. 2015 Epub 2015/09/18. 10.1089/ars.2015.6367 26377848 PMC4744882

[19] Shah PP, Beverly LJ. Regulation of VCP/p97 demonstrates the critical balance between cell death and epithelial-mesenchymal transition (EMT) downstream of ER stress. Oncotarget. 2015;6(19):17725–37. 10.18632/oncotarget.3918 25970786 PMC4627341

[20] Pan J, Zhang Q, Wang Y, You M. 26S proteasome activity is down-regulated in lung cancer stem-like cells propagated in vitro. PLoS One. 2010;5(10):e13298 10.1371/journal.pone.0013298 20949018 PMC2952619

[21] Barthelme D, Sauer RT. Origin and Functional Evolution of the Cdc48/p97/VCP AAA+ Protein Unfolding and Remodeling Machine. J Mol Biol. 2015 10.1016/j.jmb.2015.11.015 . 26608813 PMC4860136

[22] Yamamoto S, Tomita Y, Hoshida Y, Iizuka N, Monden M, Iuchi K, et al Expression level of valosin-containing protein (p97) is correlated with progression and prognosis of non-small-cell lung carcinoma. Ann Surg Oncol. 2004;11(7):697–704. 10.1245/ASO.2004.10.018 . 15231524

[23] Vij N, Fang S, Zeitlin PL. Selective inhibition of endoplasmic reticulum-associated degradation rescues DeltaF508-cystic fibrosis transmembrane regulator and suppresses interleukin-8 levels: therapeutic implications. J Biol Chem. 2006;281(25):17369–78. 10.1074/jbc.M600509200 . 16621797

[24] Min T, Bodas M, Mazur S, Vij N. Critical role of proteostasis-imbalance in pathogenesis of COPD and severe emphysema. J Mol Med (Berl). 2011;89(6):577–93. Epub 2011/02/15. 10.1007/s00109-011-0732-8 21318260 PMC3128462

[25] Liu J, Chu L, Wang Y, Duan Y, Feng L, Yang C, et al Novel peptide-dendrimer conjugates as drug carriers for targeting nonsmall cell lung cancer. Int J Nanomedicine. 2011;6:59–69. 10.2147/IJN.S14601 21289982 PMC3025585

[26] Taghavi Pourianazar N, Gunduz U. CpG oligodeoxynucleotide-loaded PAMAM dendrimer-coated magnetic nanoparticles promote apoptosis in breast cancer cells. Biomed Pharmacother. 2016;78:81–91. 10.1016/j.biopha.2016.01.002 . 26898428

[27] Yoon AR, Kasala D, Li Y, Hong J, Lee W, Jung SJ, et al Antitumor effect and safety profile of systemically delivered oncolytic adenovirus complexed with EGFR-targeted PAMAM-based dendrimer in orthotopic lung tumor model. J Control Release. 2016 10.1016/j.jconrel.2016.02.046 . 26951927

[28] Bodas M, Min T, Vij N. Early-age-related changes in proteostasis augment immunopathogenesis of sepsis and acute lung injury. PloS one. 2010;5(11):e15480 Epub 2010/11/19. 10.1371/journal.pone.0015480 21085581 PMC2981560

[29] Duong HQ, Hwang JS, Kim HJ, Seong YS, Bae I. BML-275, an AMPK inhibitor, induces DNA damage, G2/M arrest and apoptosis in human pancreatic cancer cells. Int J Oncol. 2012;41(6):2227–36. 10.3892/ijo.2012.1672 23076030 PMC3583630

[30] Gogineni VR, Nalla AK, Gupta R, Dinh DH, Klopfenstein JD, Rao JS. Chk2-mediated G2/M cell cycle arrest maintains radiation resistance in malignant meningioma cells. Cancer Lett. 2011;313(1):64–75. 10.1016/j.canlet.2011.08.022 21945852 PMC3196767

[31] Zheng YL, Kosti O, Loffredo CA, Bowman E, Mechanic L, Perlmutter D, et al Elevated lung cancer risk is associated with deficiencies in cell cycle checkpoints: genotype and phenotype analyses from a case-control study. Int J Cancer. 2010;126(9):2199–210. 10.1002/ijc.24771 19626602 PMC2950001

[32] Auner HW, Moody AM, Ward TH, Kraus M, Milan E, May P, et al Combined inhibition of p97 and the proteasome causes lethal disruption of the secretory apparatus in multiple myeloma cells. PLoS One. 2013;8(9):e74415 10.1371/journal.pone.0074415 24069311 PMC3775786

